# Feasibility and Potential Effects of Multidomain Interventions to Improve the Cognitive and Functional Well-Being of Elderly Individuals in Residential Structures: The I-COUNT Pilot Study Protocol

**DOI:** 10.3390/healthcare13161999

**Published:** 2025-08-14

**Authors:** Zaira Romeo, Eleonora Macchia, Chiara Ceolin, Maria Devita, Alessandro Morandi, Marianna Noale, Stefania Maggi

**Affiliations:** 1 Neuroscience Institute, National Research Council, 35128 Padua, Italy; zaira.romeo@in.cnr.it (Z.R.); eleonoramacchia@cnr.it (E.M.); marianna.noale@in.cnr.it (M.N.); stefania.maggi@in.cnr.it (S.M.); 2 Geriatrics Unit, Department of Medicine (DIMED), University of Padua, 35128 Padua, Italy; maria.devita@unipd.it; 3 Department of Neurobiology, Care Sciences and Society, Aging Research Center, Karolinska Institutet and Stockholm University, 17177 Stockholm, Sweden; 4 Department of General Psychology, University of Padua, 35131 Padua, Italy; 5 Department of Clinical and Experimental Science, University of Brescia, 25121 Brescia, Italy; alessandro.morandi@unibs.it; 6 Azienda Speciale Cremona Solidale, 26100 Cremona, Italy

**Keywords:** multidomain interventions, aging, mediterranean diet, functional food, cognitive stimulation, physical exercise, vaccines, pilot study

## Abstract

**Background/Objectives**: Multidisciplinary approaches spanning the physical, cognitive, and social domains of geriatric evaluation are essential to promote functional well-being and reduce the aversive consequences of aging. The main objective of the pilot study, “Multidomain Interventions to improve the COgnitive and fUNctional well-being of elderly individuals in residential sTructures” (I-COUNT), is to assess the feasibility of a 6-month multidomain intervention performed on older adults in Long-Term Care Facilities (LTCFs), compared with a group of residents following a traditional care approach. **Methods**: The intervention will involve two LTCFs in Italy and will include physical exercise and cognitive training, administered and monitored using wearable technologies, a nutritional program based on the Mediterranean diet enriched with selected functional foods, and the administration of the vaccinations recommended in the national vaccination plan. The I-COUNT study will assess the feasibility and acceptability of the defined protocol and provide information to determine the sample size for a definitive study. In relation to the potential health impact of multidomain interventions on older people living in LTCFs, the primary outcome will consider the change in microbiota composition assessed 3 months after the start of interventions, while secondary outcomes will include the evaluation of changes in selected biomarkers, physical performance, psychological health, cognitive functioning, and nutritional status at 6- and 9-month follow-up points. **Conclusions**: The I-COUNT study will allow us to assess the feasibility of delivering a multidomain intervention on elderly people. Exploratory findings on potential health effect will support the development of a larger-scale randomized controlled trial. **Trial registration number**: ClinicalTrials.gov ID NCT06820710.

## 1. Introduction

The demographic changes in recent decades have led to a significant increase in the proportion of older adults, with projections suggesting that by 2050, 16% of the global population will be aged 65 and older [1]. This trend affects many countries, particularly Italy, which has one of the highest proportions of older people in Europe, with individuals aged ≥65 years accounting for approximately 24% of the population in 2024, up from 21.5% in 2014 [2]. In this context, aging and age-related diseases represent a growing challenge for individuals, for families, and for social, economic, and health systems [3].

The consequences of aging are heterogeneous, often including a combination of clinical and subclinical conditions that contribute to frailty and diminished independence. Geriatric syndromes—multifactorial clinical conditions that do not fall into defined disease categories—result from the accumulation of impairments across multiple systems, making older adults particularly vulnerable to situational challenges [4]. This evidence underscores the multiple healthcare needs of older adults [5] and highlights the importance of identifying and adopting preventive approaches [6]. Recent studies from the “Finnish Geriatric Intervention Study to Prevent Cognitive Impairment and Disability” (FINGER) network, an international research initiative that promotes and tests multidomain lifestyle interventions aimed at preventing cognitive decline and dementia in older adults, emphasize the critical role of multidisciplinary interventions involving several specialists to provide older individuals with the most comprehensive care. These interventions address various domains of geriatric assessment, including cognitive, social, and physical aspects [7,8]. The results are encouraging, as they show that the elderly population at risk of cognitive decline benefits from multidomain lifestyle interventions, regardless of baseline characteristics [9]. Furthermore, implementing person-based approaches is essential for identifying and strengthening strategies that preserve their integrity and, most importantly, their autonomy [10].

Given these premises, particular attention should be directed to non-hospital settings, such as Long-Term Care Facilities (LTCFs), which are increasingly responsible for caring for older people across different age groups. LTCFs offer a variety of medical and personal care services to those who cannot live independently or need some type of long-term care support. To ensure successful aging, studies have been conducted to evaluate the effectiveness of physical and cognitive stimulating programs, along with nutritional interventions, aimed at preventing or mitigating frailty and disability. In particular, resistance training, combined with balance exercises, seems to prevent the deleterious effects of sarcopenia—the loss of muscle mass and strength—which is an age-related progressive condition, closely associated with the development of frailty [11,12]. Physical exercise training is also effective in improving cognitive status, physical function, and overall quality of life [13]. Similarly, dietary recommendations that align with the Mediterranean diet, emphasizing appropriate protein and caloric intake, are particularly beneficial for frail patients. In addition, the use of functional foods, containing probiotic strains and/or nutraceuticals, has demonstrated significant health benefits, contributing to disease prevention and supporting therapy [14]. Cognitive stimulation is highly recommended for people at risk of developing dementia and more generally for those in the early stages of cognitive decline. This intervention aims to improve cognitive function, enhance quality of life, and slow the progression of the disease [15]. Finally, vaccination programs are particularly important in comorbid and frail patients, as they help improve overall quality of life by reducing the risk of infections and their associated complications [16,17].

Despite these positive outcomes, the available studies often exhibit significant heterogeneity, both in terms of the population involved and the methods used to deliver the various interventions. Additionally, the feasibility and acceptability of a multidomain intervention—particularly one that incorporates new technologies to deliver and monitor these interventions among older adults residing in LTCFs—remain unclear [8].

In order to test the feasibility of a multidisciplinary intervention in older individuals living in residential structures and explore its potential effects, we designed the “Multidomain Interventions to improve the COgnitive and fUNctional well-being of elderly individuals in residential sTructures” (I-COUNT) protocol. This intervention includes a combination of selected physical activities and cognitive training, which will be administered and monitored through new technologies. In fact, physical activities have been shown to improve muscle mass, strength, and also cognition and mood, in particular among frail older adults [18,19,20]. Furthermore, cognitive training, in particular when based on new technologies and adapted to individual abilities, has been linked to improvement in memory, attention and executive functioning [21,22]. Additionally, the protocol incorporates a dietary intervention also including functional foods; dietary interventions based on the Mediterranean diet enriched with functional foods have been linked to anti-inflammatory and neuroprotective effects, contributing to gut and brain health [23,24,25]. Finally, the administration of vaccines in line with the National Vaccination Plan is suggested; in fact, vaccinations can be essential for prevention in frail populations and have been shown to reduce hospitalization and mortality in LTCFs [26,27,28]. The multidomain intervention will last 6 months and will be compared with the standard care adopted in the same LTCFs. This study is designed as a pilot and feasibility trial to evaluate whether the proposed multidomain intervention can be successfully implemented in LTCFs and to identify the necessary adaptations for a future large-scale, randomized, controlled trial. The potential health impact of multidomain interventions on older people living in LTCFs will be preliminarily evaluated considering the effect on microbiota composition and the differences in cognitive, nutritional and anthropometric status, psychological health, physical activity levels, and specific biomarkers between the intervention and control groups. Finally, we will also explore the potential impact of the multidomain intervention on participants’ perceived quality of life, as a secondary outcome.

## 2. Materials and Methods

The I-COUNT project is supported by the Italian National Recovery and Resilience Plan (PNRR) under the EU’s Next Generation EU (NGEU) program, within the Age-It initiative, which focuses on developing innovative approaches to mitigate frailty and prevent functional and cognitive decline in older adults (https://ageit.eu/wp/en/ (accessed on 16 April 2025)). Age-It identifies the most relevant challenges (e.g., demographic, biological, clinical, socio-economical, technological, and political), each of them addressed in a specific spoke. In particular, Spoke 8 of the Age-It project includes six work packages, three of which will focus on intervention studies targeting older adults in three different settings (community, hospital, and LTCF), while the other three are dedicated to the development and enhancement of technological solutions and tools to support intervention delivery, data management, and cost-effective analyses.

### 2.1. Study Design

**Figure 1:**
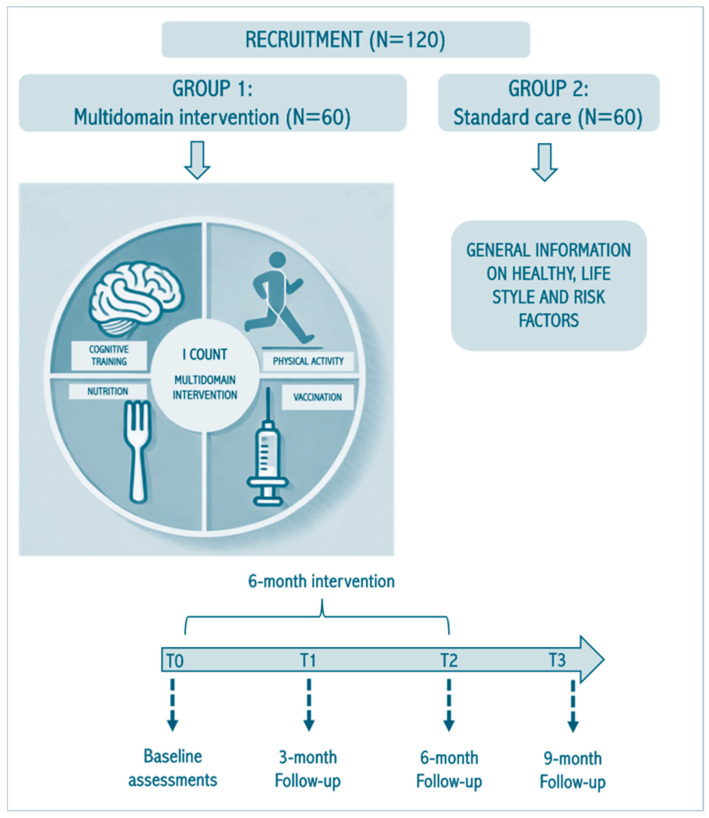
Flow diagram of the I-COUNT study. Graphical representation of the various phases of the project: recruitment, group assignment, multidomain intervention, and follow-ups.

The pilot study will follow a parallel group cluster randomized controlled trial. Randomization will be carried out at the cluster level to minimize the risk of contamination, and clusters will be defined as distinct units within LTCF residencies (i.e., separate floors or buildings, not sharing common areas or group activities, with minimal social interactions between clusters). A 6-month multicomponent intervention will be delivered and compared with usual care. Participants assigned to the multicomponent intervention will receive a 6-month program including Mediterranean diet and functional foods, physical exercise, and cognitive stimulation (see Multidomain intervention section). Participants in the intervention group will also be offered vaccinations according to the national vaccination calendar. The study protocol will include baseline evaluations (T0), a 6-month multidomain intervention, and follow-up assessments at 3 months (T1), 6 months (T2), and 9 months (T3). Participants in the control group will continue the standard care provided in their respective LTCFs, including their usual physical and cognitive stimulation activities, as well as their usual diet. Participants assigned to the control group will receive general information on healthy lifestyle and health, including risk factors. Figure 1 shows the flow diagram of the I-COUNT study protocol.

This protocol was designed in accordance with the CONSORT guidelines for feasibility and pilot studies. See the online Supplementary Materials for the CONSORT checklist figure. Participants or the public were not involved in the design of the study protocol.

### 2.2. Recruitment Process

Participants will be recruited from two Italian LTCFs: AltaVita-IRA (Padova, Italy) and Azienda Speciale Cremona Solidale (Cremona, Italy). In this pilot phase, the two participating LTCFs were selected based on established partnerships from previous collaborative research projects. Residents of the LTCFs will be recruited according to specific inclusion criteria: age ≥70 years; both sexes; residence in the identified LTCFs for at least 6 months; ability to communicate and collaborate with the research team; and Mini-Mental State Examination (MMSE) ≥18. The age threshold of 70 years was selected to focus on a population at greater risk of cognitive and functional decline, more representative of typical LTCF residents. The MMSE cut-off of ≥18 ensures the inclusion of participants with mild cognitive impairment, who retain sufficient cognitive capacity to engage in the multidomain intervention, while excluding individuals with moderate-to-severe dementia who may not safely or reliably participate in study procedures. Requiring a minimum 6-month stay in the LTCFs ensures that participants are well-adapted to the residential environment. Exclusion criteria include estimated length of stay in the LTCFs < 6 months; estimated life expectancy <6 months; previous gastrectomy or colectomy (due to potential interference with nutrient absorption and diet-related outcomes); percutaneous endoscopic gastrostomy (PEG) or nasogastric tube feeding; dysphagia; inability to undergo psychometric tests for any reason; history of psychiatric illness according to clinical anamnesis; and inability to walk, which could preclude safe participation in the physical activity component of the intervention. For each LTCF, residents will be screened for eligibility in collaboration with the facility’s staff, and eligible individuals will be invited to participate through structured in-person meetings. A total of 120 residents will be recruited, with 60 participants receiving the multidomain intervention and the remaining 60 participants assigned to the control group.

#### 2.2.1. Sample Size

The endpoint related to sample size refers to changes in gut microbiota composition between the two groups (multidomain intervention; control) assessed at 3-month follow-up compared to baseline. As suggested by Sim and Lewis to maximize precision and efficacy for pilot studies [29], a sample size of 30 individuals in the intervention group and 30 individuals in the control group is planned for each participating LTCF. This resulted in a total of 60 participants per group (120 in total). This allows for the detection of an effect size of 0.52 with 80% power and alpha = 0.05, considering a simplified approach assuming a two-sample *t*-test with equal variance (PASS 2024, version 24.0.1).

#### 2.2.2. Randomization and Blinding

Two LTCFs will participate in the I-COUNT study, and within each LTCF, two units (floors or flats) will be randomly assigned to either the intervention or control group using the SAS Plan procedure. Participants will not be informed of their group allocation until the completion of the baseline assessment. Outcome evaluators will be blinded to participants’ group assignments, and participants will be instructed not to disclose this information to the assessors. The geriatricians and neuropsychologists responsible for developing the training activities will be different from the outcome assessors. If maintaining blinding for the outcome assessors is not feasible, additional assessors will be recruited to ensure impartiality.

Data will be processed in accordance with the European Regulation on the use of personal data (679/2016) and Legislative Decree no. 196 ss.ii. 30 June 2003, as amended by Legislative Decree no. 101 of 10 August 2018, as well as Deliberation of the Privacy Guarantor no. 52 of 24 July 2008 and subsequent updates.

### 2.3. Baseline Assessments

After obtaining the informed consent from the participants, the baseline evaluations (T0) will be performed and the following data will be collected: sociodemographic characteristics, medical and pharmacological history, vaccination records, and Multidimensional Prognostic Index (MPI) [30], evaluated considering Activities of Daily Living (ADL), Instrumental Activities of Daily Living (IADL), Short Portable Mental Status Questionnaire (SPMSQ), Mini Nutritional Assessment-Short Form (MNA-SF), Exton-Smith Scale (ESS), Cumulative Illness Rating Scale (CIRS), and the number of drugs. Specifically, comorbidities and their severity will be assessed using the CIRS, and a complete list of current medications (including dosage and therapeutic indications) will be recorded for each participant. Baseline assessments will also include evaluations of nutritional, physical, and cognitive function, as well as selected biomarkers. Data collection will be performed by trained personnel, according to a predefined operating procedure; for each domain, validated instruments will be used to ensure reliability and comparability measurements.

#### 2.3.1. Dietary Assessment

Diet will be monitored over a two-week period, taking into account the menus offered by the LTCFs and assessing the type and amount of food actually consumed (food frequency questionnaire) by the study participants [31]. Staff will be trained in interview techniques and administering the food frequency questionnaire to ensure consistency.

#### 2.3.2. Body Composition and Physical Evaluation

A comprehensive whole-body bioelectrical impedance analysis (BIA) using a tetrapolar configuration will be performed by trained personnel, according to standardized procedures. The equipment will undergo daily calibration each morning, and participants will rest in a supine position for at least 5 to 10 min before the measurement begins. Appendicular skeletal muscle mass (ASMM) will be calculated using the equations developed by Sergi et al. [32]. The appendicular skeletal muscle mass index (ASMMI) will be obtained by dividing ASMM by the subject’s height in meters squared. Anthropometric parameters (i.e., body weight, height, calf and arm circumferences) will also be collected. Additional anthropometric data—including body weight, stature, and the circumferences of the calf and upper arm—will be collected. Body weight will be measured with a precision of 0.1 kg using a standard scale, with participants wearing light clothing and no footwear. If individuals are unable to stand, a hoist scale will be employed. For those who cannot maintain an upright posture, height will be estimated based on knee-to-heel length using Chumlea’s equations [33]. Body mass index (BMI) will then be derived as weight (kg) divided by height in meters squared. Calf circumference will be recorded at the widest part of the dominant leg, while the subject is either lying down or seated with the knee bent at a 90-degree angle, using a flexible measuring tape. Mid-upper arm circumference (MUAC) will be measured at the midpoint between the acromion and the olecranon on the dominant arm. To fully assess sarcopenia, both upper limb strength and physical performance will be evaluated. Handgrip strength will be tested using JAMAR TM Plus+ electronic dynamometers (Patterson Medical, Green Bay, WI, USA) by qualified medical personnel. Each hand will be tested three times, and the final grip strength will be calculated as the average of the best results from both the dominant and non-dominant hands.

Physical performance will be evaluated by the 6-min walking test and Short Physical Performance Battery (SPPB), which includes standing balance tests (side-by-side stands, semi-tandem test, and tandem test), gait speed measurement, and timed chair stands test, generating scores between 0 and 12, with higher scores indicating better physical performance [34].

#### 2.3.3. Neuropsychological and Psychological Assessments

Standardized neuropsychological batteries and tests will be administered to all participants before starting the training to assess their cognitive functioning; assessments will be carried out by trained staff. Global cognitive functioning will be measured through the Mini Mental State Examination (MMSE) [35]. Addenbrooke’s Cognitive Examination Revised (ACE-R) battery [36] will also be administered to assess cognitive functioning in different domains (e.g., global flexibility, attention/orientation, memory, fluency, language and visuospatial abilities). In addition, the WHOQOL-BRIEF will be administered to assess four domains assumed to represent the quality of life construct: physical domain, psychological domain, social relationships domain, and environmental domain [37]. The Cognitive Reserve Index questionnaire (CRIq) will be administered to quantify the cognitive reserve accumulated by individuals throughout their lives [38]. Finally, the Geriatric Depression Scale-GDS [39], Depression Anxiety Stress Scales-DASS-21 [40], and the Perceived Stress Scale-PSS [41] will also be administered. Sleep quality will be evaluated with the Pittsburgh Sleep Quality Index (PSQI, [42]).

#### 2.3.4. Biomarkers

A blood sample will be obtained from the study participants for the assessment of blood counts, lipid, glucose and metabolic profiles, vitamin D, inflammatory parameters, and a urine sample. A stool sample for gut microbiota analysis will be collected within three days from enrollment, following a predefined procedure to ensure uniform sample integrity through controlled timing, storage, and handling to preserve microbiota integrity. Metagenomic analysis will be performed to generate microbiome profiling data together with metadata associated with the investigated samples.

### 2.4. Multidomain Intervention

The multidomain intervention will involve different experts, such as geriatricians, neuropsychologists, physiotherapists, exercise science experts, clinical nutrition experts, and digital coaches. Participants will be informed of their group allocation after baseline data collection.

Data will be collected through a computer interface specifically designed for the study (REDCap) (https://project-redcap.org/ (accessed on 16 April 2025)) [43,44].

#### 2.4.1. Nutritional Intervention

The nutritional history of the participants will be evaluated at the beginning and at the end of the intervention, and then during follow-up, by collecting food habit data with a food frequency questionnaire.

Participants in the intervention group will be encouraged to follow a Mediterranean diet, in particular to consistently use extra virgin olive oil as the main fat source and to have a regular intake of nuts, and they will also consume functional foods, in particular sourdough-containing bread fortified with a vegetable matrix rich in polyphenols (e.g., olive leaves) and one probiotic vegetable (e.g., artichokes). Before starting the treatments, the functional foods will be evaluated for their functional properties (bioactive compounds, probiotics) [45]. Sourdough biotechnology has been demonstrated to improve digestibility and promote healthy microbiota metabolism at the colon level [46,47]. Moreover, olive leaves are considered a cheap source of polyphenols such as secoiridoids, flavonoids, phenylpropanoids, caffeic acid, tyrosol, and hydroxytyrosol [48]. The other approach will consider the use of a vegetable matrix enriched with Lacticaseibacillus paracasei IMPC2.1 probiotic strain, produced according to the innovative and patented procedures reported by Lavermicocca et al. [49,50], and authorized by the Italian Ministry of Health. Human feeding studies demonstrated that this probiotic strain is able to reach the gut in adequate amounts when delivered through vegetable matrices such as artichokes and table olives [51,52,53]. The final products will be characterized for their polyphenol content, and in vitro gastrointestinal digestion, using static and/or dynamic systems, will be employed to assess their potential health benefits. All the activities will be carried out with the companies in charge of developing the functional foods envisioned in this proposal.

#### 2.4.2. Physical Intervention

After individual evaluation, a physiotherapist or an exercise science expert will prescribe appropriate exercises for ad hoc supervised small group sessions. Each rehabilitation session will last approximately 40 min and will include a warm-up with stretching exercises (~5 min), a cool-down (~5 min), and rest periods as needed, three times a week. Exercise intensity and complexity will be tailored to each participant’s functional capacity, assessed at baseline, and progressively adapted based on performance. Based on the international guidelines for physical exercise in the old population, and considering the VIVIFRAIL Multicomponent Physical Exercise Program to Prevent Frailty and the Risk of Falls (available from https://blogs.bmj.com/bjsm/2021/03/06/vivifrail-a-multi-component-physical-training-program-to-prevent-weakness-and-falls-in-people-over-70-years/ (accessed on 16 April 2025)), participants will be asked to perform the following:

- Endurance exercises with muscle strengthening of the lower and upper limbs, such as getting up from a chair or bed, front and side step-ups, lifting the lower limbs, lifting water bottles, etc. The physiotherapists or exercise science experts will adapt the type of exercises to the participant’s abilities;
- Static and dynamic balance exercises of increasing difficulty, tailored to the patient’s initial performance level and subsequent improvements (e.g., exercises to maintain a standing position on a flat surface, load transfer, proprioceptive exercises on an unstable surface or balance board, maintaining balance on monopodal support, semi-tandem walking on a straight line, walking with small objects, etc.). If necessary, the physiotherapist or the exercise science expert will use tools to reduce the risk of falling during treatment;
- Exercises for aerobic activity will include walking for a number of minutes appropriate to the patient’s baseline functional performance level, following the patient’s subsequent progress during the sessions.

Exercises will be performed in small groups (3–5 participants) under continuous supervision. The VIVIFRAIL protocol, which allows for safe progression according to individual performance, will be applied, with a gradual increase in difficulty, adapted to the participant’s progress and under close observation. Each training session will include rest periods and monitoring of fatigue, pain, or changes in health status before and during sessions. Several of the following strategies will be implemented to prevent falls during exercise: At the baseline assessment, physical function and balance will be evaluated by a physiotherapist or exercise specialist prior to starting the program, to tailor exercises to each participant’s capacity, and sessions will be led by trained professionals (physiotherapists or exercise scientists) in small groups. Furthermore, participants will be monitored before and during each session for signs of fatigue, instability, or clinical changes, and sessions will be suspended or modified if safety concerns arise.

Accelerometers and other wearable sensors (smartwatches) will be used to monitor the physical activity performed. Minimally invasive wearable sensors will be used to monitor the performed activities through (a) inertial-based technologies for gait/posture analysis and (b) surface EMG-based devices for muscle activity profiling (imbalance evaluation).

Health problems and symptoms will be evaluated by the physiotherapist or the exercise science expert before starting each session. If a participant reports a significant change in health before or during the exercise, the geriatrician will be consulted. Vital signs (heart rate, blood pressure, and oxygen saturation) will be recorded at the beginning and the end of each session. Physical exercise training sessions will be stopped in the presence of (a) resting systolic blood pressure >200 mmHg or diastolic blood pressure >100 mmHg; (b) resting heart rate >120 beats/min or <40 beats/min; (c) increased heart rate 90% of maximum age during exercise; (d) oxygen saturation <90% in ambient air; (e) unusual or severe dyspnea; (f) oppressive pain or discomfort in the chest, pain in the left arm, indigestion or discomfort in the stomach; (g) palpitations; (h) severe dizziness, fainting or fainting sensation; (i) decrease in diastolic blood pressure by 20 mmHg during exercise; (j) increase in systolic blood pressure to 250 mmHg or diastolic blood pressure to 115 mmHg during exercise; or (k) decrease in oxygen saturation <88% in ambient air during exercise. The clinical conditions of the participants and the levels of intensity and duration of the exercise will be re-evaluated.

#### 2.4.3. Cognitive Intervention

In the week prior to the start of the intervention, the neuropsychology team will organize face-to-face meetings to introduce the cognitive training program to the participants and to promote adherence. During these meetings, participants will also become familiar with the online platform used for the training. A cognitive intervention based on the cognitive stimulation approach will be delivered by expert neuropsychologists twice a week (48 sessions in total, approximately 30 min each). Residents will be divided into small groups (3–5 people) with similar cognitive levels, and a computerized tool will be used for the intervention (REmote stimulation for COgnitive DEcline (RECODE)). RECODE was developed by researchers at the University of Padova (https://osf.io/eqgyn/?view_only=fa14090e85f7424abf73bf583871b529 (accessed on 16 April 2025)) with the aim of stimulating multiple cognitive domains (i.e., attention, working memory, visual memory, language, orientation, and executive functions) through exercises of increasing difficulty. Each participant will use a personal laptop during the RECODE session, starting at the basic level and progressing to the next level only when they have achieved 80% accuracy in a given domain, ensuring a personalized intervention. RECODE is designed for cognitive enhancement in older people with mild-to-moderate cognitive impairment and has an age-friendly graphical interface with an avatar that interacts with the participant. Sessions will follow a fixed structure, as follows: (1) a short introduction and guidance by the neuropsychologist; (2) execution of exercises from different domains; and (3) immediate feedback from the platform and the neuropsychologist. Adherence will be monitored, and motivational strategies (group discussions, reinforcement, support from caregivers or LTCF’s staff) will be implemented to sustain participation.

Physical and cognitive interventions will be administered in a smart environment, with technological solutions including mobile and wearable devices, ensuring high acceptability by the users. In particular, smartwatches and tablets with SIM cards and pre-loaded specific applications will be used for physical and cognitive training and for monitoring specific conditions (risk of falls). Health professionals responsible for the different interventions will explain to study participants and to their family members the use of the devices (basic functions, such as turning the device on and off, and opening specific applications).

#### 2.4.4. Vaccinations

Participants in the intervention group will be encouraged to receive appropriate vaccinations according to the national vaccine calendar; for adults over 65 years of age, the national vaccine program recommends the influenza, pneumococcal, herpes zoster, and diphtheria–tetanus–pertussis vaccinations (Ministero della Salute Italiano. Italian immunisation schedule, https://www.epicentro.iss.it/vaccini/piano-nazionale-vaccini-2023–2025 (accessed on 27 June 2025)). Vaccine doses will be provided by the Department of Preventive Medicine, Vaccine Office, National Health Service, on prescription from the general practitioners.

### 2.5. Follow-Up Assessments

Three months after the beginning of the multidomain intervention (T1), a stool sample will be collected for the analysis of the gut microbiota; this is the only assessment planned at T1. At the end of the intervention (6 months, T2) and three months later (9 months, T3), the assessments will be repeated. Furthermore, anthropometric data and blood and stool samples will be collected, together with assessments of physical and cognitive performance, at both T2 and T3. While the 6-month time point will allow us to evaluate the immediate effects of the intervention, the 9-month follow-up is intended to assess the durability of any observed effects, to evaluate whether possible improvements are maintained, attenuated or lost once the intervention is discontinued.

### 2.6. Retention

In order to promote continued participation in the study, a set of strategies will be implemented, as follows: participants will receive regular reminders to attend training sessions (printed notes, announcements by the staff); study staff (nurses, LTCFs coordinators) will encourage participants to follow the suggested activities and to use the smartwatch provided; and, in addition to participants, family members and caregivers will also be informed about the project and the activities. Furthermore, sessions will be adapted, when necessary, and their schedules will be managed flexibly to minimize burden or fatigue. Participants will receive continuous positive reinforcement (verbal encouragement and group support) to help maintain motivation and adherence over time.

With regard to follow-up visits, at the end of each visit, the date of the next visit will be set and instructions given; reminders, such as printed notes, will be delivered to participants’ rooms or handed to them during meals by staff members. Short in-person meetings or group announcements will be organized by staff members to reinforce upcoming sessions or follow-up assessments. Indirect reminders will also be sent to family members, when appropriate, via phone calls or emails from the facilities’ contact person.

### 2.7. Analytical Approach

#### 2.7.1. Outcomes

The feasibility of the multidomain intervention will be assessed by considering the acceptability and the participants’ adherence to the interventions. In particular, feasibility outcomes will include the following:

- Recruitment and retention rates over the study period;
- Adherence metrics (attendance at training sessions, completion of cognitive modules, dietary compliance);
- Technological acceptability, assessed via user feedback and actual usage data (wearable sensors, digital apps);
- Completeness and quality of data collected across domains;
- Operational challenges, including staffing, logistics, and data management within LTCFs.

The primary health outcome of this short-term pilot study is the evaluation of changes in gut microbiota composition and the differences between the two groups (multidomain intervention and control groups) at the 3-month follow-up point (T1). The gut microbiota has emerged as a central player in the gut–brain–muscle axis, and growing evidence suggests it may influence metabolism, inflammation, muscle function, and neurocognitive processes, making it a valuable biomarker in multidomain interventions for older adults [54].

Secondary outcomes were selected to reflect the domains of cognitive and functional well-being referenced in the study title and central to the I-COUNT intervention, and will include the assessment of changes in the following:

- Gut microbiota composition and differences between the two groups at follow-up at 6 and 9 months (T2 and T3);
- Hemogram, electrolytes, cholesterol, triglycerides, blood glucose, vitamin D, and other parameters and differences between the two groups at 6- and 9-month follow-ups (T2 and T3);
- Additional biomarkers (including ESR, high-sensitivity CRP, albumin, transferrin, IL-6, BDNF, FT3/FT4, s-RAGE, irisin, IGF1, ghrelin) and differences between the two groups at 6- and 9- months follow-up (T2 and T3);
- Physical performance (SPPB) and differences between groups at 6- and 9-month follow-ups (T2 and T3);
- General and domain-specific cognitive functioning (mNTB, ACE-R) and differences between groups at 6- and 9-month follow-ups (T2 and T3);
- Psychological health (GDS, PSS, DASS), sleep quality (PSQI) and differences between groups at 6- and 9-month follow-ups (T2 and T3);
- Number of falls and number of hospital admissions and differences between groups at 6- and 9-month follow-ups (T2 and T3);
- Nutritional, anthropometric status (MNA-SF, BMI, waist circumference), and body composition, and differences between the groups at 6- and 9-month follow-ups (T2 and T3).

#### 2.7.2. Adverse Events

An adverse event, defined as any unintended unwanted outcome, including abnormal laboratory results, symptoms or illnesses, falls, and hospital admissions that occur during the intervention, whether or not related to the intervention, will be reported.

#### 2.7.3. Statistical Analyses

Descriptive statistics will be calculated in relation to feasibility measures (recruitment and retention rates over the study period, adherence metrics, technological acceptability, completeness of data collected and operational challenges).

Continuous variables will be compared between groups through mixed models, with random effects at the cluster level and treatment group as fixed effects. The potential health impact of multidomain interventions will be estimated considering changes over time for continuous variables analyzed through mixed models for repeated measures, with group and time, and group x time interaction as fixed effects, and cluster and patients as random effects.

The continuous variables considered in comparison between groups for fecal microbiota analyses will include measures of species richness (α-diversity), the relative abundance of specific bacterial taxa, and the representation of genes involved in key bacterial metabolic pathways. The effects of the intervention on the overall fecal microbiome composition will also be assessed comparing intervention and control groups on different time points with Bray–Curtis dissimilarity on principal coordinate analysis.

Data available for participants with complete data will be considered, but also an intention-to-treat (ITT) approach with imputation for missing data will be applied.

Results with a *p*-value < 0.05 will be considered statistically significant. Analyses will be performed using SAS software version 9.4 (SAS Institute, Cary, NC, USA).

## 3. Discussion

Aging is characterized by an increasing vulnerability to various chronic conditions, making it the primary risk factor for multimorbidity. The gradual accumulation of deficits, which notably intensifies with advancing age, is a key indicator of declining resilience and widespread imbalance across multiple bodily systems [55]. Moreover, some age-related diseases (such as dementia) have a multifactorial etiology [56]. A multimodal approach that addresses multiple risk factors and mechanisms simultaneously may be necessary to achieve optimal preventive effects.

The present pilot and feasibility study aims to evaluate the implementation of multidomain, person-based interventions for mitigating the adverse consequences of aging and improving the cognitive and functional well-being of older individuals. We propose the I-COUNT pilot protocol, which integrates physical and cognitive training, and adherence to the Mediterranean diet and to appropriate vaccinations, as a novel multicomponent intervention for older people living in LTCF settings. The I-COUNT pilot study aims to assess the feasibility of the defined protocol and to provide information to determine the sample size for a definitive study. Furthermore, this study will also provide preliminary data on cognitive, functional, and biological outcomes.

Although there is encouraging evidence regarding the effectiveness of individual programs (e.g., diet, physical exercise, or cognitive stimulation), guidelines to standardize and regulate these types of interventions are still lacking, especially in out-of-hospital care settings, where the specific equipment differs from those available for hospitalized patients. Furthermore, multidomain interventions have recently been shown to be more effective than single-domain interventions in improving frailty status, muscle mass and strength, and overall physical functioning [57]. This makes the I-COUNT project particularly valuable, as it combines interventions known to prevent disability and to improve general health and quality of life, offering a specific protocol for implementation within LTCF residences in Italy.

The domains addressed in the I-COUNT pilot protocol are crucial for preserving current abilities or delaying the onset of decline in older adults. Moreover, interventions targeting one domain often have positive effects on different domains as well. For example, the reduction in frailty score and improvements in cognitive status are significantly mediated by the composition and functionality of the gut microbiota in individuals following the Mediterranean diet [54]. The beneficial effects of physical exercise on cognitive status are also well documented [58,59]. Finally, vaccination, together with other interventions (e.g., diet, physical training) can contribute to healthy aging, also preserving the independence of older adults [60]. In our opinion, multidomain interventions represent the best option to preserve the health of older adults and protect them from frailty. We expect that positive changes might be observed in each domain during the intervention, as well as improvement in perceived quality of life and psychological status.

All the measures collected before the training and during the follow-ups (including biomarkers, physical performance, neuropsychological and psychological data) will enable us to assess the feasibility of the multidomain intervention protocol, analyze its effectiveness in detail, and to create a biobank for future studies on risk profiles related to the development of cognitive and functional disability. As a pilot and feasibility study, I-COUNT is not designed to test efficacy or effectiveness in a definitive sense but to explore whether the intervention can be successfully delivered in LTCF settings, and to identify operational, technological, and procedural aspects that may require refinement. These outcomes will provide critical information to refine intervention components, improve training procedures, and optimize data collection protocols. Importantly, data from this pilot will be used to calculate effect sizes and variability estimates, which are essential for accurate sample size calculation in a future multicenter randomized controlled trial. Moreover, qualitative and process evaluations will help us better understand barriers and facilitators to implementation in real-world aged care settings.

The present study has some limitations: first, only the feasibility of interventions is assessed, but future research should also evaluate the actual costs and benefits in the real world; second, the interventions adopted in the I-COUNT protocol (RECODE, technological instruments, etc.) need to be validated.

## 4. Conclusions

Overall, the I-COUNT pilot protocol offers a personalized and comprehensive program for older adults living in LCTF residences. If the feasibility and acceptability of the multidomain intervention are proven in this pilot study, the exploratory findings will support the design of a larger-scale trial study. The findings of this study will be potentially relevant for clinicians, healthcare providers, and caregivers, and could guide policymakers and stakeholders in recognizing the urgent need for innovative interventions in caring for older adults. This research provides a foundation for further studies in clinical practice and health policies, with the goal of building a more sustainable and effective healthcare system for aging populations.

## Data Availability

No new data were created or analyzed in this study. Data sharing is not applicable to this article.

## Supplementary Materials

The following supporting information can be downloaded at https://www.mdpi.com/article/10.3390/healthcare13161999/s1, CONSORT checklist figure. Ref. [61] was cited in the Supplementary Materials.

## Abbreviations

The following abbreviations are used in this manuscript:

ACE-R	Addenbrooke’s Cognitive Examination Revised
ADL	Activities of Daily Living
ASMM	Appendicular Skeletal Muscle Mass
ASMMI	Appendicular Skeletal Muscle Mass Index
BIA	Bioelectrical Impedance Analysis
BMI	Body Mass Index
CIRS	Cumulative Illness Rating Scale
CRIq	Cognitive Reserve Index questionnaire
DASS	Depression Anxiety Stress Scale
ESS	Exton–Smith Scale
GDS	Geriatric Depression Scale
IADL	Instrumental Activities of Daily Living
I-COUNT	Multidomain Interventions to improve the COgnitive and fUNctional well-being of elderly individuals in residential sTructures
ITT	Intention-to-treat
LTCFs	Long-Term Care Facilities
MMSE	Mini-Mental State Examination
MNA-SF	Mini Nutritional Assessment-Short Form
MPI	Multidimensional Prognostic Index
MUAC	Mid-upper arm circumference
PNRR	National Recovery and Resilience Plan
PSQI	Pittsburgh Sleep Quality Index
PSS	Perceived Stress Scale
RECODE	Remote stimulation for Cognitive DEcline
SPMSQ	Short Portable Mental Status Questionnaire
SPPB	Short Physical Performance Battery
WHOQOL-BRIEF	World Health Organization Quality of Life scale
